# Brightness cues affect gap negotiation behaviours in zebra finches flying between perches

**DOI:** 10.1098/rsos.240007

**Published:** 2024-06-05

**Authors:** Emma Borsier, Helen Sanders, Graham K. Taylor

**Affiliations:** ^1^ Department of Biology, University of Oxford, Oxford OX1 3SZ, UK

**Keywords:** zebra finch, gap negotiation, obstacle avoidance, phototaxis, bird flight, guidance

## Abstract

Flying animals have had to evolve robust and effective guidance strategies for dealing with habitat clutter. Birds and insects use optic flow expansion cues to sense and avoid obstacles, but orchid bees have also been shown to use brightness cues during gap negotiation. Such brightness cues might therefore be of general importance in structuring visually guided flight behaviours. To test the hypothesis that brightness cues also affect gap negotiation behaviours in birds, we presented captive zebra finches *Taeniopygia guttata* with a symmetric or asymmetric background brightness distribution on the other side of a tunnel. The background brightness conditions influenced both the birds’ decision to enter the tunnel aperture, and their flight direction upon exit. Zebra finches were more likely to initiate flight through the tunnel if they could see a bright background through it; they were also more likely to fly to the bright side upon exiting. We found no evidence of the centring response that would be expected if optic flow cues were balanced bilaterally during gap negotiation. Instead, the birds entered the tunnel by targeting a clearance of approximately one wing length from its near edge. Brightness cues therefore affect how zebra finches structure their flight when negotiating gaps in enclosed environments.

## 1. Introduction

Birds and insects rely on vision to guide flight through cluttered environments, displaying many convergent similarities in the mechanisms that they use. Many of the best-known visual guidance strategies of birds and insects involve the use of optic flow [1–11], which refers to the movement of visual contrast across the visual field that results from relative motion between an observer and its environment. One such flight behaviour, first described in honeybees *Apis mellifera* [5] and later reported in budgerigars *Melopsittacus undulatus* [6], involves steering to balance the optic flow between the left and right visual hemispheres. This response naturally produces centred flight along corridors with symmetrically striped walls, but its behavioural consequences could be highly variable in complex natural environments such as forests. Indeed, subsequent research has found no evidence for an optic flow balancing response in hummingbirds *Calypte anna*, whose lateral steering behaviour instead depends on the vertical extent of visual features in the environment [12].

Vegetated environments are often densely cluttered, so the optic flow that they produce is both rich in information and complex to interpret. Birds and insects nevertheless manage to find their way quickly through clutter [11,13–15], even when presented with conflicting visual information [16], so their guidance strategies must be robust to such complexity. One possibility is that they achieve this by integrating optic flow cues with other kinds of visual information. For example, the visual system that guides perching in budgerigars relies specifically on edge detection [17]. Likewise, Harris’ hawks *Parabuteo unicinctus* have been reported both to direct their gaze at the edges of obstacles they are avoiding [18] and to steer towards a clearance of approximately one wing length from an obstacle’s edge [19]. Nevertheless, in cluttered environments such as forests, there may be many edges to attend to, so it is reasonable to ask whether there are simpler heuristics that might also be used to guide flight.

Several studies have modelled obstacle avoidance in birds and insects as a gap negotiation behaviour [11,13–15], which means that rather than treating obstacles as repellers in a sense-and-avoid strategy, as is typically used in engineering contexts [19], the clearance between them is treated as an attractor [11,13–15] in a sense-and-approach strategy. While the two treatments might be functionally equivalent under certain circumstances, they differ mechanistically—particularly in relation to the sensory cues that they may be expected to rely upon. For instance, whereas optic flow expansion cues are evidently suitable for detecting upcoming obstacles in a sense-and-avoid strategy, brightness cues provide a simple and direct indicator of the open space between obstacles that could be used in a sense-and-approach strategy. Consistent with this expectation, Baird and Dacke [20] found that orchid bees *Euglossa imperialis* employ a strategy based on using relative brightness cues to detect and negotiate gaps in their environment. When presented with apertures of different shapes or varying background lighting conditions, the orchid bees flew towards the point of greatest brightness within the aperture.

It is not yet known whether birds employ a similar strategy to detect and negotiate gaps during flight, but it is reasonable to expect that they might—particularly given how strongly migratory birds are attracted to artificial light at night [21–23]. Birds also tend to fly towards windows when trapped inside a room, and birds that fly into windows outdoors often appear to be flying towards a patch of brightness (e.g. crashing into a window because they are flying towards a second window that can be seen through the first). Gap negotiation behaviours are likely to be of general importance to perching birds (Passeriformes) owing to their use of vegetated habitats. For example, the zebra finch *Taeniopygia guttata* inhabits open sclerophyll woodland, open shrublands and arid grassland environments with scattered trees and shrubs [24]. We therefore treat the zebra finch as a model for obstacle avoidance in species adapted to flight through open vegetated environments, noting that other mechanisms may be important in species adapted to flight through densely vegetated environments.

Here, we ask specifically whether brightness cues influence the gap negotiation behaviours of zebra finches in flight. To answer this question, we recorded the flight behaviour of captive birds presented with varying background brightness conditions viewed through the aperture of a narrow flight tunnel between two adjoining aviaries (electronic supplementary material, movie S1). Our results show that whether and how the birds chose to fly through the tunnel was strongly influenced by the background lighting conditions, shedding light on how birds may use simple brightness cues to guide their flight behaviour.

## 2. Material and methods

### 2.1. Animals

**Figure 1.**
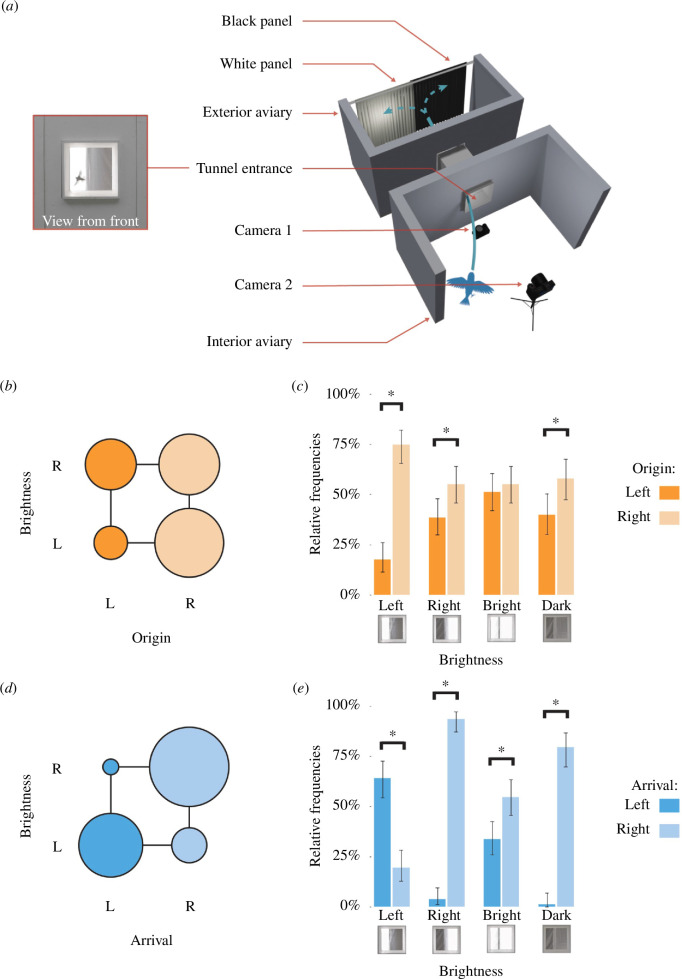
The flight of zebra finches through a tunnel was influenced by the background brightness distribution visible through its aperture. (*a*) Representation of the experimental set-up (not to scale), showing Camera 2’s view of the tunnel, illustrating a bird exiting to the bright side (see electronic supplementary material, video S1). (*b,d*) Bubble plots showing the number of flights originating (*b*) or arriving (*d*) on the left or right side of the aviary, split according to whether the background was bright to the right or the left. Circle area is shown as proportional to the count. (*c,e*) Bar charts showing the frequency of origin (*c*) or arrival (*e*) on the left and right sides of the aviary, split according to background brightness condition; error bars display 95% confidence intervals computed using the Agresti–Coull method. The icons below the plots illustrate the appearance of the background behind the aperture, and frequencies are shown relative to the total number of flights recorded under each brightness condition (i.e. including those where the side of origin or arrival was ambiguous). L, left; R, right.

We used a captive-bred population of *n* = 24 adult zebra finches including 13 males and 11 females (wingspan = 185.8 ± 4.0 mm; mean ± s.d.). We conducted the experiments in a large aviary with multiple perches, comprising separate interior and outside parts connected by a square-section tunnel of width 0.33 m and length 0.58 m through which the birds could fly freely (figure 1*a*; electronic supplementary material, movie S1). Each part of the aviary measured up to 5 m in width, 2 m in depth and 2.7 m in height. Feeders were set up in the exterior aviary, while nest boxes and a water dispenser were set up in the interior aviary to encourage the birds to fly back and forth through the tunnel. Perches and nest boxes were evenly distributed between the left and right sides of the aviaries to encourage birds to make use of both sides.

### 2.2. Experimental set-up

The indoor aviary was painted white and was lit with a mixture of 6500 and 4000 K flicker-free LED lighting; the outdoor aviary was painted dark brown on its internal walls and received natural light through its mesh frontage and right side looking out from the tunnel; the interior of the connecting tunnel was painted white. We attached panels of white voile fabric and/or black blackout cloth to the mesh frontage of the exterior aviary, which we hung up and took down each day. We began by hanging a pair of white panels on the inside of the mesh opposite the tunnel’s exit, covering the entire area visible through the tunnel from anywhere in the interior aviary (figure 1*a*). This allowed natural light to stream through the white fabric, providing a bright background that hid any visual landmarks located outside the aviary. To vary the spatial distribution of the incoming natural light, we then hung black panels on the outside of the mesh behind the white panels to create four alternative experimental conditions: (i) dark symmetric; (ii) bright symmetric; (iii) bright to the left; and (iv) bright to the right (electronic supplementary material, figure S1).

Because the white fabric was a light-transmissive voile material whereas the black fabric was a blackout material, the dark and bright panels always had a contrast ratio of at least 3:1. This exceeds the contrast sensitivity of other diurnal birds such as the domestic pigeon *Columba livia domestica* and the common starling *Sturnus vulgaris* [25], so the contrast between the light and dark panels should always have been saturated independent of the ambient lighting levels. Furthermore, although the light intensity measured at the tunnel exit varied from 500 to 8000 lux depending on cloud cover, the light intensity at the tunnel entrance was never more than 400 lux. The white-painted tunnel walls would therefore always have been intermediate in brightness between the dark and bright backgrounds that we presented. The unavoidable presence of an upright structural element supporting the aviary mesh meant that the brightness distribution could not be made perfectly uniform under the bright-symmetric condition and could not be made perfectly mirror symmetric under the bright-to-the-left and bright-to-the-right conditions (electronic supplementary material, figure S1). While this is an inconvenient structural feature of the aviary that we used, the experimental design and statistical analysis are nevertheless robust to this asymmetry.

### 2.3. Experimental design

We recorded the birds’ flight behaviour over 12 days, recording two sessions per day lasting from 1.0 to 1.5 h each. We alternated the presentation of the asymmetric bright-to-the-left and bright-to-the-right conditions between sessions on 7 of the 12 test days (electronic supplementary material, table S1). We presented either the dark-symmetric or bright-symmetric condition during both test sessions on the remaining 5 test days (electronic supplementary material, table S1). This experimental design ensured that the presentation of the different test conditions was uncorrelated with environmental factors such as the time of day, position of the sun or order of the sessions.

### 2.4. Video data and analysis

We filmed *n* = 419 flight trajectories of zebra finches using the tunnel to travel from the interior to the exterior aviary, using two synchronized cameras recording 120 frames per second at 3840 × 2160 pixels (Z-CAM E2, Shenzhen, China). The cameras were mounted below or behind the entrance to the tunnel (figure 1*a*) and were aligned to its centreline. We reviewed the video data to record the side of origin and side of arrival of each flight (left versus right, scored as ambiguous if the bird vacillated before entering the tunnel), together with the lateral projection (φ) of the bird’s point of entry as viewed from the camera placed below the tunnel’s entrance, defined on the interval −1 < φ < 1 where φ = 0 corresponds to a centred entry (see electronic supplementary material, figure S2). We used the video data to classify the birds into distinct morphotypes to verify that the effects we describe were not being driven by a small subset of individuals (see electronic supplementary material, supplementary methods). We use the results of this classification to demonstrate the consistency of our findings across morphotypes graphically below. However, as we were only able to identify 19 distinct morphotypes within our population of *n* = 24 individuals and could not always reliably determine which morphotype was represented in each video, we are limited to treating flight trajectories recorded from the same birds as independent data points. We analysed the data statistically using R (v. 4.0.3) and R Studio (v. 1.3.1093) with the packages PropCIs [26] and diptest [27].

## 3. Results

Although the population sex ratio was approximately balanced (54% males), there was a strong sex bias in the flights that we recorded (88% males), with males approximately six times more likely to fly through the tunnel than females (odds ratio: 6.2; Wilson score 95% CI: 2.7, 14.5).

**Figure 2.**
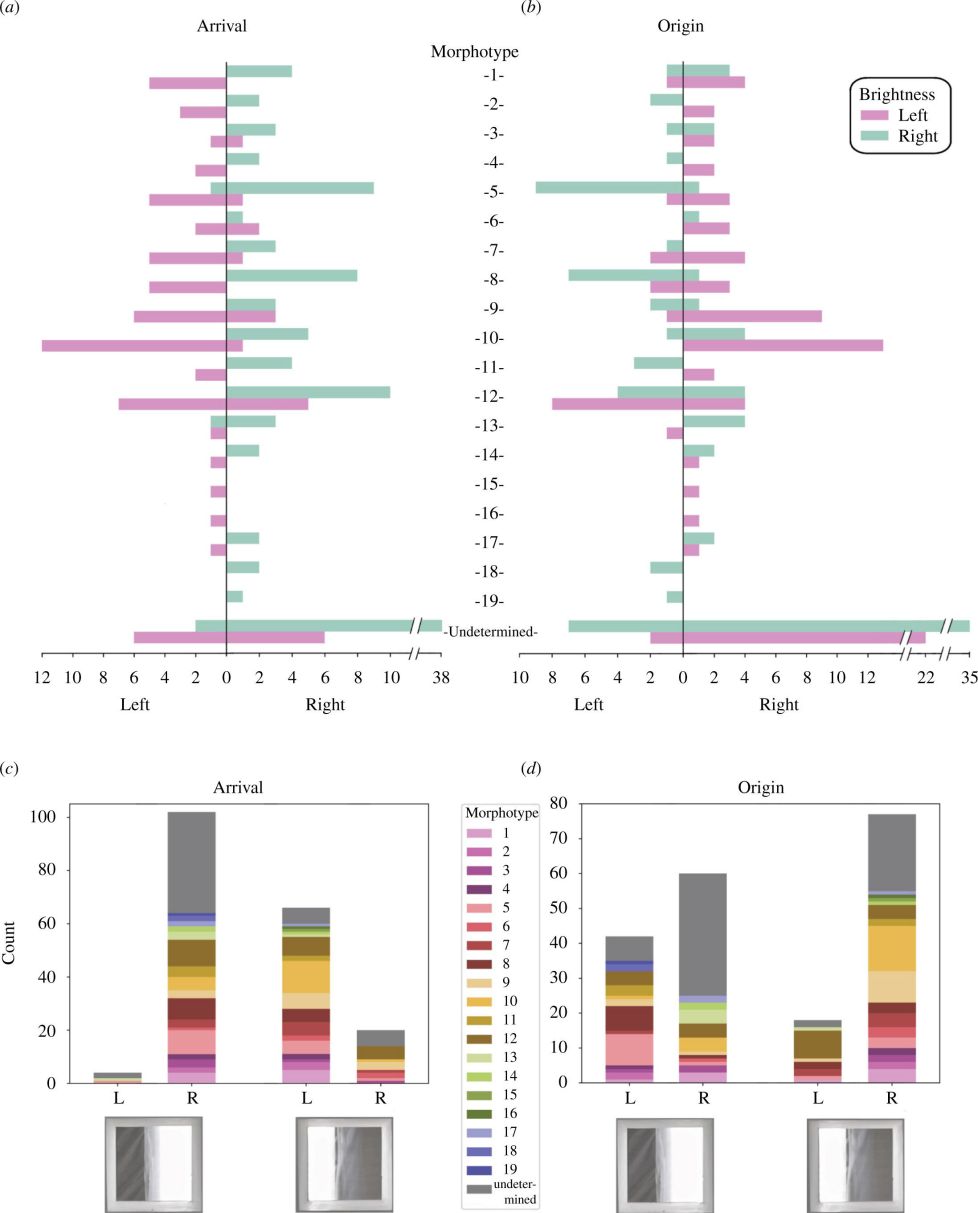
Brightness condition affects the sides of origin and arrival in most of the birds represented in this study. (*a,b*) Bar charts showing, for each of the 19 morphotypes and one indeterminate category, the number of flights that: arrived on (*a*) or originated from (*b*) the left or right side of the aviary, depending on whether the background was bright to the right (green) or bright to the left (pink). (*c,d*) Bar charts stacked by morphotype, showing the total number of flights that arrived on (*c*) or originated (*d*) from the left and right sides of the aviary depending on background brightness condition. L, left; R, right.

Most of the flight trajectories that we recorded arrived on the right side of the exterior aviary (70%; Wilson score 95% CI: 65, 74%; *n* = 368 unambiguous records), reflecting an overall preference for landing on the perches located on the right side of the exterior aviary, opposite the access door and adjacent to a mesh side wall (table 1; figure 1*d*). Despite this lateral bias, there was nevertheless a highly significant relationship between brightness condition and arrival side (*χ* ^2^(3) = 154.63, *p* < 0.001), with the birds switching their preferred arrival side from right to left when the background was bright to the left (table 1; figure 1*e*). Considering the two asymmetric brightness conditions together, the birds were seven times more likely to arrive on the bright than the dark side of the aviary (odds ratio: 7.0; Wilson score 95% CI: 4.6, 10.7). This pattern was displayed by most of the 19 morphotypes that we identified, as well as by the indeterminate category (figure 2*a*,*c*). It follows that the birds used brightness cues to select their arrival side, and hence to select the goal of their flight.

**Table 1.** Frequency distributions of the side of origin and arrival for each of the four brightness conditions. The side of origin or arrival was sometimes ambiguous (?) in flights with *S*-shaped trajectories.

brightness condition	origin			arrival		
	left	right	?	left	right	?
bright	56	60	3	40	65	14
dark	35	51	2	1	70	17
left	18	77	8	66	20	17
right	42	60	7	4	102	3
total	151	248	20	111	257	51

Most of the flight trajectories originated from the right side of the interior aviary (62%; Wilson score 95% CI: 57, 67%; *n* = 399 unambiguous records), reflecting an overall preference for perches located on the right of the interior aviary (figure 1*b*). This bias was displayed under all four test conditions (table 1; figure 1*c*), but there was still a statistically significant relationship between side of origin and brightness condition (*χ* ^2^(3) = 20.567, *p* < 0.001). Specifically, the birds’ tendency to originate from the right was amplified when the background was bright to the left rather than the right (odds ratio: 3.0; Wilson score 95% CI: 1.6, 5.7; figure 1*c*). This same effect was observed across many of the 19 morphotypes that we identified, as well as by the indeterminate category (figure 2*b*,*d*). Under this test condition, the tunnel aperture would have appeared bright when viewed from the right and dark when viewed from the left, so this result is consistent with the hypothesis that the birds were preferentially targeting brighter gaps. Hence, although any lateral asymmetry in destination brightness affected the birds’ side of origin in the opposite direction to their side of arrival, both effects are consistent with a common underlying mechanism favouring flight towards a brighter destination.

The birds’ tunnel entry points did not appear to be centred under any of the test conditions: instead, the birds tended to enter the tunnel on either the left or the right, and only rarely in the middle (figure 2*a*). A Hartigan’s dip test confirmed that the entry point distribution was non-unimodal under each of the two symmetric test conditions (bright: *D* = 0.072, *p* < 0.001; dark: *D* = 0.066, *p* = 0.005) and under the asymmetric test condition with the background bright to the right (*D* = 0.057, *p* = 0.009), where the first two modes were estimated at entry points φ ≈ ±0.3 (table 2). For the asymmetric condition with the background bright to the left, there was no evidence of non-unimodality (*D* = 0.025, *p* = 0.95), with the first mode estimated at an entry point of φ ≈ 0.3 (table 2). In summary, under all four test conditions, the birds tended to enter the tunnel to one side or the other at φ ≈ ±0.3, rather than centring themselves at φ = 0, and this tendency was displayed by many of the morphotypes that we identified (electronic supplementary material, figure S3).

**Table 2.** Mode(s) of entry points φ of the zebra finches as they entered the tunnel under each of the four brightness conditions.

brightness condition	entry point mode(s)
bright	−0.33, 0.35
dark	−0.25, 0.28
left	0.27
right	−0.24, 0.27

**Figure 3.**
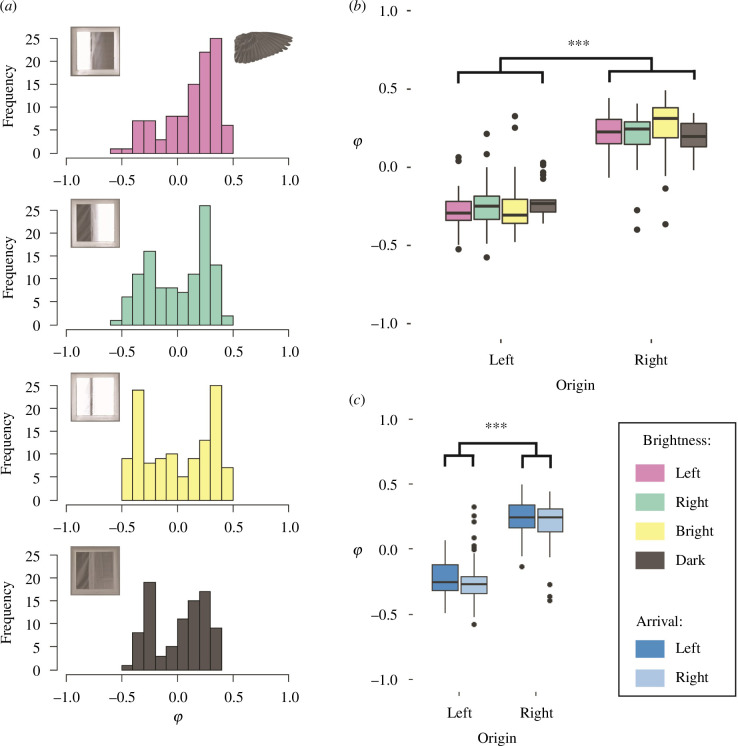
The entry point of zebra finches into the tunnel depends on the side that they arrive from. (*a*) Histograms plotting the lateral projection (φ) of the bird’s point of entry into the tunnel under each of the four background brightness conditions, as illustrated by the inset camera views (bright to the left, bright to the right, bright symmetric and dark symmetric). A zebra finch wing is depicted for scale, showing that the birds typically target a clearance of approximately one wing length upon entry. (*b,c*) Boxplots of entry point φ by side of origin, grouped according to: (*b*) background brightness condition and (*c*) side of arrival.

The lateral projection of the birds’ point of entry into the tunnel was dependent on their side of origin (two-sample *t*‐test: *t*_397_ = −30.8, *p* < 0.001): birds originating from the left tended to enter to the left of centre (φ = −0.25 ± 0.15; mean ± s.d.; *n* = 151), whereas birds originating from the right tended to enter to the right of centre (φ = 0.23 ± 0.15; mean ± s.d.; *n* = 248). This was consistently observed across most of the 19 morphotypes that we identified, as well as by the indeterminate category (electronic supplementary material, figure S4). However, whereas the birds’ side of origin was itself related to brightness condition (see above), we found no significant interaction between the effects of the side of origin and brightness condition on the birds’ entry point when comparing only the two asymmetric brightness conditions (two-way ANOVA: *F*_1173_ = 0.722, *p* = 0.397; figure 3*b*). In contrast, the birds’ arrival side had a significant effect on their point of entry into the tunnel when controlling for their side of origin (two-way ANOVA: *F*_1345_ = 4.98, *p* = 0.026; figure 3*c*). The birds’ point of entry into the tunnel was therefore determined by where they were flying from and to; each of which in turn relates to the background brightness condition (see above).

## 4. Discussion

The results of this simple flight experiment (figure 1; electronic supplementary material, movie S1) demonstrate that the flight trajectories of zebra finches are influenced by the brightness properties of the background towards which they are flying. Specifically, the distribution of background brightness seen through an aperture affects: (i) a bird’s propensity to take off and fly through an aperture (figure 1*b,c*), and hence the side from which the bird is most likely to enter the aperture (figure 3*a*); and (ii) where the bird flies to upon exiting the aperture (figure 1*d,e*). Although our birds preferred to enter the tunnel from the right side, they were three times more likely to do so if the background viewed through its aperture was brighter to the left than the right (figures 1*b* and 2*d*). Hence, as the left side of the background was visible from the right side of the aviary, and vice versa, it follows that the birds were more likely to take off and fly through the tunnel if the tunnel appeared as a bright aperture from their original perching point. Likewise, when exiting the tunnel, the birds were more likely to fly towards the brighter side of the aviary (figures 1*d* and 2*c*).

Taken together, our results suggest that the goal of the birds’ flight behaviour was decided before take-off and was structured phototactically in relation to brightness cues. Our results do not allow us to confirm whether brightness cues are used to guide reactive steering during flight, but they do imply that brightness is used in the birds’ path planning before take-off. Because clutter usually correlates negatively with brightness in vegetated environments [20] including in the sclerophyll woodlands and shrublands inhabited by wild zebra finches, we suggest that phototactic behaviour may aid birds in finding clear paths through cluttered environments. Furthermore, as certain motion-selective neurons in birds are most responsive to dark targets moving against light backgrounds [28], such phototactic behaviour may also aid in foreground object detection.

Our findings from zebra finches are therefore broadly consistent with the findings from orchid bees by Baird and Dacke [20], who noted that in cluttered environments such as forests, brightness cues can ‘provide information about the point of greatest clearance in an aperture, the relative size of an aperture as well as the clearest path’ [20]. However, whereas orchid bees appear to steer their flight using background brightness cues, targeting the brightest point of an unevenly lit aperture, we found no evidence that the lateral projection of the bird’s entry point was affected in detail by the background brightness distribution (figure 3*a*), except insofar as this brightness distribution determined the side of the aviary from which they entered the tunnel (figure 3*b*). That said, our experimental design differs from that of Baird and Dacke [20], who used a different brightness distribution and differently shaped apertures.

Our zebra finches did not aim for the centre of the tunnel aperture (figure 3*a*), as might have been expected had they been balancing the optic flow generated by the tunnel’s aperture. Instead, they entered the tunnel from the side on which they were perched before take-off (figure 3*b,c*), resulting in a bimodal entry-point distribution (figure 3*a*). The birds turned tightly around the near corner of the tunnel aperture, aiming at an entry point located at φ ≈ ±0.3 and hence approximately 0.1 m from the near side of the tunnel aperture. This behaviour left them with a clearance of approximately one wing length from the tunnel wall (figure 3*a*), consistent with results from Harris’ hawks, which also aim for a clearance of approximately one wing length when steering around obstacles [19]. It follows that our observations do not support the hypothesis that zebra finches centre their flight by balancing the optic flow between their left and right visual hemispheres upon entering a tunnel. It is important to note, however, that our observations on where the birds entered the tunnel do not preclude the possibility that the birds might have adjusted their trajectory after entering the tunnel in a centring response like that observed in bumblebees [4], honeybees [5] and budgerigars [6].

Concerning other limitations of our findings, it is worth noting that males were over-represented in our flight data, and that we were only able to separate the population into 20 distinct morphotypes as opposed to being able to distinguish all *n* = 24 individuals on every flight. Individual honeybees [29] and pigeons [15] can exhibit distinct biases for a given side during gap selection, and budgerigars are known to adopt idiosyncratic flight trajectories for repetitive flight tasks [30], so formally controlling for between-individual variation in the flight trajectories could provide further information on the effect of contrast for this experiment. Nevertheless, our results clearly show that zebra finches use brightness cues to structure their flight when negotiating gaps in closed environments, and that the same qualitative effect is present in multiple individuals. Behavioural experiments that have been set up specifically to test only the effects of optic flow cues may therefore be missing important parts of the picture when it comes to assessing how birds and insects negotiate clutter in natural environments.

## Data Availability

Data and relevant code for this research work are available at Dryad [31], and have been archived within the Zenodo repository [32]. The data used in the statistical analysis are provided as a .csv file in the electronic supplementary material [33]. A sample video from one of the cameras is uploaded as movie S1. The raw video data are stored in a local archive and will be made available upon reasonable request.
